# Split spawning realigns coral reproduction with optimal environmental windows

**DOI:** 10.1038/s41467-018-03175-2

**Published:** 2018-02-19

**Authors:** Taryn Foster, Andrew J. Heyward, James P. Gilmour

**Affiliations:** Australian Institute of Marine Science, Indian Ocean Marine Research Centre, University of Western Australia, 39 Fairway Street, Crawley, 6009 WA Australia

## Abstract

Split spawning in coral populations occurs when gamete maturation and mass spawning are split over two consecutive months. While split spawning has been observed at many reefs, little is known about the frequency and significance of these events. Here we show that split spawning occurred frequently and predictably over a decade at Scott Reef. Split spawning overlays the biannual spawning pattern in the region and occurs when the full moon falls in the first week of the usual spawning month, or the last week of the previous month. Additionally, in split years most species have their main spawning event after a 13-month lunar cycle, in the month following the usual spawning month. Without split spawning, spawn dates would shift by ~10 days each year to occur outside of optimal environmental windows. Our results suggest that split spawning is driven by a disconnect between lunar and seasonal cues, and is analogous with a ‘leap year’ in coral reproduction, realigning spawning dates with favourable conditions for reproduction.

## Abstract (web summary)

Corals usually undergo single mass spawning events, however, occasionally they split reproductive effort across two months. Here, Foster et al. use 10 years of data to determine the drivers and timing of split spawning, showing that these events realign spawning with optimal environmental conditions.

## Introduction

The timing of coral spawning is complex, varying not only between locations and species, but also from year to year. At some reefs, including the Great Barrier Reef (GBR) and several Western Australian (WA) reefs, many species and colonies spawn within hours of one another on certain nights of the year. This mass spawning is thought to enhance fertilisation success while also increasing survival through predator satiation^1,2^. Since its discovery in the early 1980s^3^, this phenomenon has intrigued researchers, with many studies investigating how corals synchronise their gamete release. The triggers for spawning involve a variety of environmental signals, including daylight hours, water temperature^4,5^, wind speed^6^, moonlight^7^, tides^8,9^ and hours after sunset^10^, with cues operating on increasingly fine temporal scales. Earlier studies on broadcast spawning suggested that synchrony was a result of large environmental fluctuations. In this model, populations that do not experience large fluctuations, such as corals on equatorial reefs, would exhibit less synchrony^11^. Many studies broadly support this hypothesis^11–14^. However, other work has shown that relatively small changes to environmental conditions may trigger spawning^5^ and there are also examples of multi-specific spawning synchrony at some low latitude reefs^15–17^. At some reefs, where spawning of the coral community is less synchronised, sequential spawning events of species assemblages occur over several months within a spawning season^11,14,18,19^. Protracted spawning or multiple spawning seasons may also be more common^13,20–24^ than the original observations of mass spawning on the GBR suggested. The oceanic reef systems off the northwest shelf of WA have two discrete mass spawning events per year, the first in autumn (usually March) and the second in spring (usually October), with several species but few colonies participating in both events^17,25^.

Within a spawning season, split spawning of the same species across consecutive months has been widely noted (Supplementary Table 1), both in regions where high levels of multi-specific synchrony occur and where more protracted spawning is the norm^3,8,10,16,17,23,24,26–33^. On the GBR, split spawning usually occurs when the full moon falls in the first half of the month in spring^10^, but can also occur when the full moon falls in the second half of the month (22nd of October and 20th of November)^8^. Pharoah and Willis^26^ tracked long-term patterns of split spawning for the central GBR. They recorded split spawning when the full moon fell in the first half of the month and hypothesised that spawning was tracking lunar patterns (12 or 13 lunar months) in order to maintain reproduction within favourable conditions. Note, however, that the data underlying the discussion of these patterns in ref. ^26^ were never published in the primary literature. In Western Australia, there are single records of split spawning occurring both when the full moon occurs early in the month (Dampier^24^ and Scott Reef^23^) and late in the month (Abrolhos Islands^28^), but no long-term records or an understanding of the frequency of split spawning on individual reefs. Across regions, there does not appear to be a consistent relationship between the timing of the full moon and the occurrence of split spawning, and this relationship is probably unique to each reef. Additionally, it is unknown what proportions of colonies spawn in each month of a split spawning event and thus it is unknown how significant these events are for reef renewal.

Unlike other causes of temporal reproductive isolation, such as protracted and biannual spawning, split spawning is not an annual feature but occurs periodically. It is important to understand the cycles of split spawning to improve the predictability of spawning events to inform; coral restoration efforts, studies of coral reproduction^17^, management initiatives aimed at reducing anthropogenic impacts during significant periods of spawning^34^ and to project future shifts in reproduction and spawning arising from climate change^35,36^. We tracked coral spawning at Scott Reef over a decade to determine when spawning was split over 2 months. We also investigated what proportion of the population was spawning in the ‘usual’ spawning month versus the following month in a split year and whether there were associated variations in fecundity and egg size. Finally, we analysed the temperature anomalies in the 6 months prior to spawning (the period when corals were undergoing gametogenesis) to determine whether temperature variation was correlated with split spawning. Our findings show that split spawning is occurring frequently and predictably, according to the timing of the full moon, in both spawning seasons at Scott Reef and that the main spawning event in a split year is the second month, not the ‘usual’ spawning month. Split spawning occurs after 13 lunar months instead of 12 lunar months, having the effect of realigning the spawning date within the optimal environmental window for successful fertilisation and recruitment.

## Results

### Spawning patterns from 2007 to 2016

**Fig. 1:**
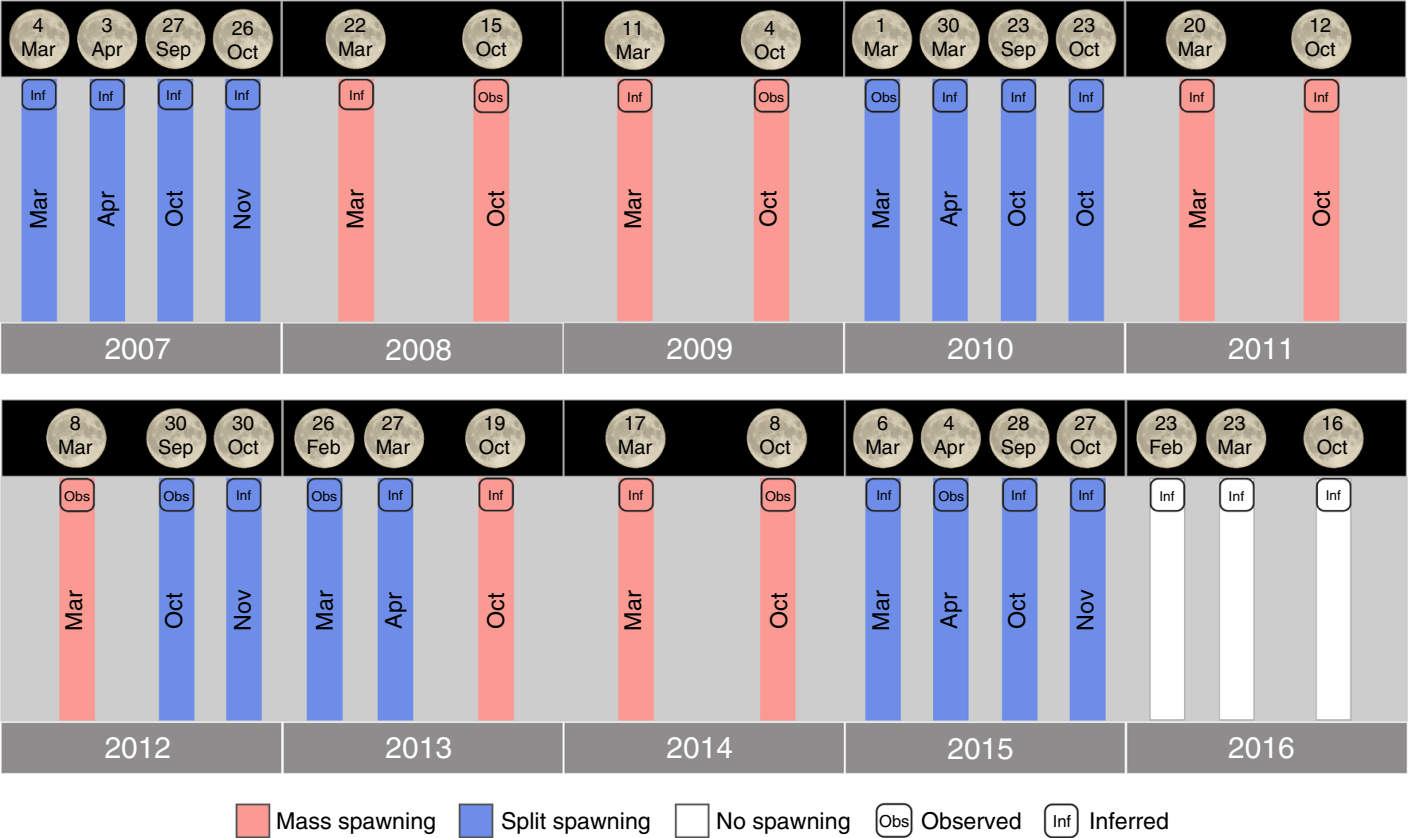
Coral spawning records for Scott Reef from 2007 to 2016. Spawning was either observed in situ or inferred from egg presence/absence and egg pigmentation around predicted spawning dates. When the full moon occurred in the middle (8th–22nd) of the usual March or October spawning month, then single-mass spawning occurred. However, when the full moon occurred early, in the first week of the usual spawning month, or the last week of the previous month, then split spawning occurred. The only exception to this rule was the mass spawning event after an early full moon on the 4th October in 2009. Full moon dates are given in the moon icons above the bars. Pink bars indicate mass spawning and blue bars indicate split spawning. The white bars in 2016 indicate no spawning due to a severe coral bleaching event. More detail on these spawning records are presented in Supplementary Table 2

Split spawning at Scott Reef occurred approximately every 2–3 years over the 10-year period from 2007 to 2016 (Fig. 1). Split spawning overlayed the biannual spawning pattern at Scott Reef, where spawning events occur in both autumn (March) and spring (October). In mass spawning years, spawning occurred in March and October (2008, 2009, 2011 and 2014), while in split spawning years autumn spawning was split between March and April and spring spawning was split between October and late October/November (2007, 2010 and 2015). In some years (2012 and 2013) split spawning occurred in only one of the seasons. Note that the data summarised in Fig. 1 is presented in more detail in Supplementary Table 2.

Mass spawning during a single month occurred if the full moon fell in the middle (~8–22nd) of the usual spawning month (March and October). However, if the full moon occurred early, that is near the start of the usual month (<8th) or near the end (>22nd) of the previous month (February and September), then split spawning occurred. Of the 26 spawning events studied, the only exception to this rule was the mass spawning in October 2009 following an early full moon on the 4th of October (Fig. 1).

**Fig. 2:**
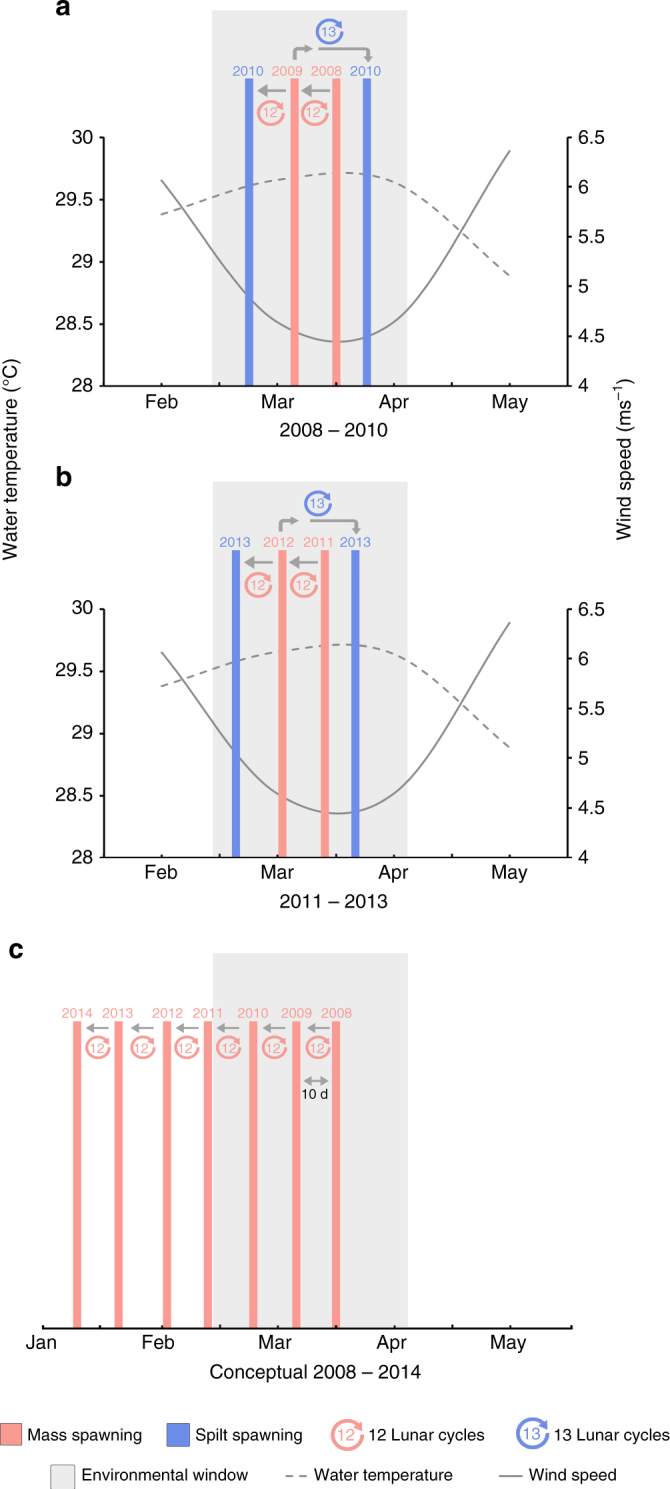
Autumn spawning dates and lunar cycles in relation to the optimal environmental window. Mass spawning events are shown with pink bars and split spawning events are shown with blue bars. **a**, **b** The empirically observed autumn spawning realignment cycles that occurred due to split spawning in 2010 (**a**) and 2013 (**b**), maintaining spawning in the environmental window (warm water temperatures and low wind speeds). Water temperatures and wind speeds for Scott Reef are the mean monthly values obtained from the Hadley Centre Sea Surface Temperature data set from 1990 to 2000 and the NOAA Earth Systems Research Laboratory from 1968 to 1996 respectively. **c** The hypothetical shift in the spawning date, out of the optimal environmental window, if spawning occurred every 12 lunar months without realignment. For example, if spawning occurred on the 30th of March in 2008 (far right pink bar) and then successively every 12 lunar months, then the spawning date would shift earlier by ~10 days each year (grey arrows), occurring on the 24th of January in 2014

In mass spawning years gamete release occurred after 12 lunar months (autumn 2008, 2009, 2011, 2012; Fig. 2a, b). However, in split years, the first spawning event occurred after 12 lunar months and the second event occurred after 13 lunar months (autumn 2010 and 2013; Fig. 2a, b). This 13-month cycle realigned spawning to occur within optimal temperature and wind conditions (environmental windows) at Scott Reef. Spawning dates were shown to shift out of the optimal environmental window in autumn, by ~10 days each year, in the absence of split spawning realignment, i.e. on (theoretical) continuous 12-month lunar cycles (Fig. 2c).

In 2016, a split spawning was predicted in autumn (March and April) and a mass spawning in spring (October). However, a mass coral bleaching event occurred in autumn and water temperatures peaked in both early March and early April, reaching ~33 °C (with daily means of ~31 °C) at ~6 m depth, and these peaks coincided with the predicted spawning dates (Supplementary Fig. 1). Consequently, no eggs were observed around the predicted second spawning date in early April and most colonies were severely bleached at this time. Similarly, most colonies had died and there were no eggs visible in the few survivors around the predicted dates of mass spawning in October 2016.

### 6-month temperature anomalies

**Fig. 3:**
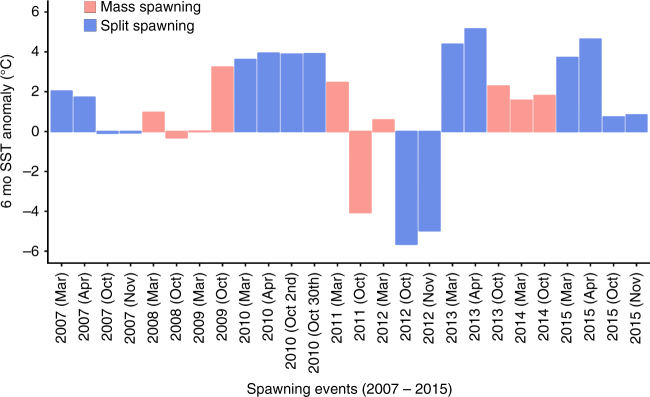
Cumulative sea surface temperature anomalies for the 6-months prior to each coral spawning event at Scott Reef from 2007 until 2015. Mass spawning events are shown with pink bars and split spawning events are shown with blue bars

Across years and in both seasons, there was no correlation between the 6-month temperature anomalies and split or single spawning events. Split spawning occurred during years with the largest positive (spring 2013) and largest negative (spring 2012) 6-month temperature anomalies, as well as during relatively neutral years (spring 2007 and spring 2015) (Fig. 3). Similarly, single-mass spawning events occurred in years when there were positive (spring 2009, autumn 2011, spring 2013, 2014), negative (spring 2011) and neutral (autumn and spring 2008, autumn 2009, autumn 2012) 6-month temperature anomalies (Fig. 3).

### Split spawning and reproductive output from 2008 to 2010

**Fig. 4:**
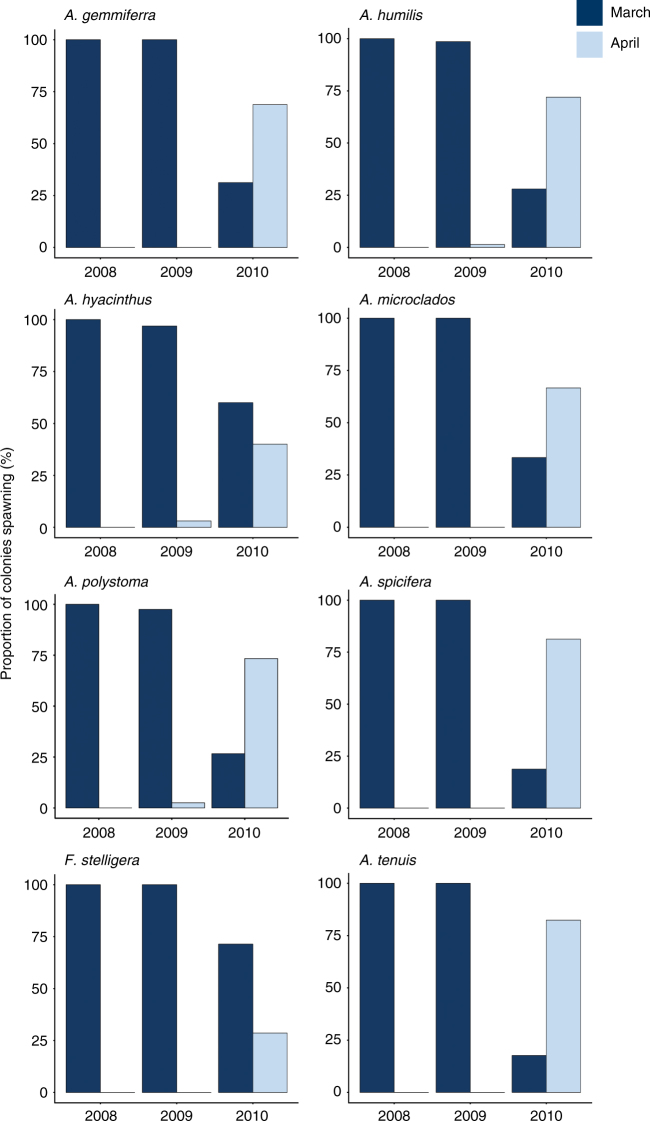
Proportions of corals spawning in autumn at Scott Reef from 2008 to 2010. Bars show the proportion of coral colonies spawning in March versus April in mass spawning years in 2008 and 2009, compared to a split spawning year in 2010 at Scott Reef. Proportions are for eight coral species and the sample sizes for each species and year are presented in Supplementary Table 3

Both 2008 and 2009 were mass spawning years, with spawning occurring in a single month during the autumn and spring spawning seasons. In autumn 2008 and 2009, almost all colonies spawned in March and not in April, with the exception of <5% of *Acropora humilis, A. hyacinthus* and *A. polystoma* colonies in April 2009 (Fig. 4). In contrast, in 2010 spawning was split over 2 months in both seasons. In autumn of 2010, all of the eight species studied had at least 25% of colonies spawning in April, however the proportion of colonies spawning during each month varied among the species (Fig. 4). Most species (*Acropora gemmiferra, A. humilis, A. microclados, A. polystoma, A. spicifera* and *A. tenuis*), had a higher proportion of their colonies (up to 80%) spawning in April compared with March. Only *Favia stelligera* and *A. hyacinthus* had smaller proportions (25 and 40%) of colonies spawning in April compared with March.

There was little evidence of split spawning affecting the reproductive output of colonies, as either the number or size of eggs at maturation. There were generally 6–8 eggs per polyp in all of the seven *Acropora* species and the range was ~4–12 eggs (Supplementary Fig. 2). Within all of the species, there were no significant differences in the egg count per polyp between mass and split spawning years (Supplementary Fig. 2). Similarly, with the exception of *A. microclados* and *A. spicifera*, there were no significant differences in mean maximal egg size between mass and split spawning years (Supplementary Fig. 3). Egg sizes for *Acropora* species were ~600 to 700 µm, but were smaller in *F. stelligera* (~300 µm).

## Discussion

Split spawning occurred frequently (~every 2–3 years) at Scott Reef during both spawning seasons (autumn and spring). Spawning was split when the full moon fell early (either in the first week of the usual spawning month or in the last week of the previous month), whereas single-mass spawning occurred when the full moon fell in the middle of the usual spawning month. This is in contrast to the GBR where split spawning occurred when the full moon fell in the first half of the month^10^, and highlights that corals in different regions may be exposed to a different combination of cues and/or respond differently to a similar combination of cues^13,26,37^. Split spawning at Scott Reef overlayed the biannual spawning pattern common in this region^22,23,25^. Spawning was split in both seasons in most split years, but in 2 years (2012 and 2013), spawning was split in one season and not the other. This pattern supports the annual realignment theory proposed in Pharoah and Willis^26^, with the annual realignment of spawning dates with environmental windows, occurring in each season. Annual realignment occurred independently in assemblages of autumn and spring spawners at Scott Reef because they are reproductively isolated and considered separate spawning populations. Existing evidence suggests that each assemblage spawns consistently in the same season each year and that very few colonies can spawn during both seasons, largely precluding interbreeding between assemblages^17,25^. This was evident with split spawning occurring in spring 2012 and then in autumn 2013, indicating spring realignment in 2012 and autumn realignment in 2013 (Fig. 1).

Spawning times coincide with favourable environmental conditions, such as warm water temperatures and low wind conditions^3,5,6,8^. If spawning occurred every 12 lunar months, spawning times would slowly shift (~10 days each year) to occur outside of optimal environmental windows (Fig. 2c). Over the course of just 6 years, ‘autumn’ spawning would shift from March to occur in January (Fig. 2c) and ‘spring’ spawning would shift from October to August. In January and August wind speeds are higher (~6 ms^−1^) than during observed spawning times in the optimal environmental windows (3.5–4.5 ms^−1^), and in August the water temperature also drops to around 26 °C (compared with the optimal 28–30 °C). Spawning during cooler and windier months would likely reduce the success of fertilisation as well as larval survival, settlement and post-settlement survival. However, in split spawning years the cycle is realigned (with environmental windows), with spawning occurring after 13 lunar months every 2 to 3 years, maintaining spawning activity within favourable conditions for fertilisation and larval survival (Fig. 2a, b). Accordingly, the majority of the species studied here had a higher proportion of colonies spawning in the second month in the split spawning year (although both months had significant spawning events, with ≥20% of colonies spawning). Further research is required to determine why the whole community does not shift in a split year, but this is probably due to individual differences in gametogenesis and responses to environmental cues.

There is very little information on the natural variations in both the onset of gametogenesis and gamete maturation rates within a species, and no studies have connected environmental drivers to maturation synchrony and subsequent split spawning. Early work on gametogenic cycles reported that gametogenesis became more synchronous as development proceeded^38–40^. However, there is at least one record of rapid egg development in corals in the oceanic Kimberley region (Ashmore Reef), where mature, pigmented eggs were observed in February and September (1 month earlier than predicted spawn dates) and it was hypothesised that this was due to warmer than normal water temperatures^17^. Interestingly, these observations were not recorded in a split spawning year, but in 2011 during the marine heatwave event in WA^41^.

Cumulative temperature anomaly analyses highlighted that unusually high temperatures were not driving split spawning. Elevated water temperatures can increase the rate of development of both gametes^38,42,43^ and embryos^44–46^, suggesting that the water temperatures in the 6 months prior to spawning, when corals were undergoing gametogenesis, would influence developmental rates and consequently spawning times. However, there was no relationship between temperature anomalies and split spawning events at Scott Reef. This lack of correlation and the cyclical nature of our split spawning records, suggest that split spawning events occur regularly and predictably, in response to the timing of the full moon and its alignment with other spawning cues. Nonetheless, our results do not preclude temperature from being involved in split spawning. It is likely that seasonal changes in temperature interact with other cues and variations in onset of oogenesis (discussed further below) to drive split spawning. Finer scale temperature analyses (higher temporal and spatial resolution) tracked against temporally fine scale (monthly) monitoring of gamete development, would help to determine the role temperature plays in development and spawning time in the context of split spawning.

The drivers of split spawning are likely a combination of a disconnect between lunar and seasonal cues and variation in the initiation and rate of oogenesis among colonies. Oogenesis in *Acropora* corals at Scott Reef takes approximately 4–6 months^25^. Small eggs have been observed in colonies of several tagged *Acropora* species at Scott Reef ~4–6 months prior to spawning. However, at that time eggs were visible in some colonies and not others (within species known to participate in the next spawning event), indicating that they had not yet initiated oogenesis^25^. In addition to variation in the onset of oogenesis, individual colonies are also likely to differ in their rate of egg development, due to variations in their energy reserves. In a ‘normal’ mass spawning season, environmental cues likely override these variations and spawning occurs in a single month. However, in a split spawning season or year these variations in onset and development, in addition to differences in environmental cues (i.e. changes in the combinations of seasonal and lunar cues as the spawning cycle shifts out of the optimal environmental window), could cause some colonies in the population to spawn in the following month. The ~10-day shift in spawning date over a 12-month lunar cycle is a result of the 11-day difference between a lunar year (354 days) and a solar year (365 ¼ days). When the full moon occurs early (either the last week of the previous month or the first week of the usual spawning month), corals receive the lunar cue to spawn when seasonal signals indicate that it is too early for spawning and/or coral gametes are not quite mature enough for spawning. This disconnect between the lunar cue and seasonal cues/gametogenic development prevents some (or most) of the colonies from spawning until the following month and is potentially the driver behind the cyclical split spawning we have observed.

Split spawning had no apparent effect on individual colony reproductive output at Scott Reef, with most species showing no difference in egg number and size between split and mass spawning years or seasons. However, the consequences of split spawning could be more significant for community reproduction, since split spawning temporally isolates reproduction among conspecifics. This routine temporal reproductive isolation reduces the density of colonies interbreeding within a spawning event, and subsequently reduces the likelihood of fertilization. The implication for populations in a healthy state with a high density of adult corals is perhaps negligible, but may be far greater on reefs experiencing recurrent severe disturbances. The extreme water temperatures and coral bleaching during 2016 at Scott Reef provide an acute example of how a disturbance can affect colony fecundity and density, reducing the likelihood of fertilisation and larval production. The regime of disturbances to which many coral reefs are now being exposed is significantly reducing coral cover^35,36,47,48^, which subsequently reduces reproduction and recruitment success^49,50^. The combination of geographical isolation, increased temporal reproductive isolation and frequent disturbances may push populations more quickly towards a tipping point at which the density of conspecific colonies is so low that fertilisation is compromised and few larvae are produced to facilitate recovery.

Split spawning occurs on frequent and predictable cycles at Scott Reef, and is usually associated with an early full moon (either the first week of the usual spawning month or the last week of the previous month). Although both months in a split year had significant spawning activity, the majority of the species had higher proportions spawning in the second month (i.e. a month later than the ‘usual’ month of spawning). We propose that split spawning is driven by a disconnect between lunar and seasonal spawning cues and appears to function analogously to a ‘leap year’ in coral reproduction, realigning spawning with favourable environmental conditions for fertilisation and larval survival. Our findings have important implications for management (particularly in regions subject to development), research activities on coral reefs, coral restoration efforts and understanding the impacts of anthropogenic climate change and other chronic disturbances that are currently re-structuring coral reproduction.

## Methods

### Study site and spawning observations

Scott Reef is an isolated system of reefs located on the edge of the continental shelf ~270 km off the northwest Australian coastline (14°04′S, 121°46′E; Supplementary Fig. 4). The Ashmore reef system (~240 km to the north) and the Rowley Shoals (~400 km to the south) are the closest coral reefs to Scott Reef. Field samples were collected at six long-term monitoring locations across the reef system. Spawning observations were collected during field trips from 2007 until 2016 (Supplementary Table 2). Spawning dates were determined directly as observations of in situ spawning and spawn slicks on the water surface, or inferred based on egg pigmentation and the proximity to full moon dates (mass spawning generally occurs 7–9 days following the full moon in March and October at Scott Reef). In this study we refer to years in which spawning was split over 2 months as ‘split spawning’ and years in which spawning was not split, as ‘mass spawning’^26^.

### 6-month temperature anomalies

Corals at Scott Reef typically take 4–6 months to undergo gametogenesis^25^. Temperatures during these months could impact rates of gametogenesis and subsequently, spawning date. To investigate whether split spawning events were driven by seawater temperature anomalies, the sum of the temperature anomalies for the 6 months prior to spawning were calculated. Extended Reconstructed Sea Surface Temperature (ERSST v5) records for Scott Reef from NOAA were used to determine mean monthly temperatures from 2007 to 2015 (2016 was excluded because no spawning occurred due to mass coral bleaching). The monthly means were compared to the extended monthly means (1854-2015) also derived from ERSST v5 (NOAA), to determine the anomaly for each month.

### Field samples

Eight species of scleractinian coral (*Acropora gemmiferra*, *Acropora humilis*, *Acropora hyacinthus*, *Acropora microclados*, *Acropora polystoma*, *Acropora spicifera*, *Acropora tenuis* and *Favia stelligera*) were sampled from up to six sites at Scott Reef. Approximately (median) 16 replicate colonies were sampled per species, per year, but sample sizes varied among species and years (see Supplementary Table 3 for sample sizes). Colonies were sampled 2 to 5 weeks prior to the predicted mass spawning dates in autumn (March/April) in 2008 and 2009 (mass spawning years) and 2010 (split-spawning year). Only sexually mature (>20 cm diameter) colonies were sampled and three branches were collected from each colony. Branches were collected from the centre of the colony to avoid sterile colony margins. During field sampling, colonies were examined in situ to score the stages of egg development. Scores were based on both the size and pigmentation of visible eggs within the polyp (see Supplementary Fig. 5 for pictures). Eggs were scored according to the following observations; score 1: large pigmented (red or pink) eggs were clearly visible within the polyp, indicating that the colony would participate in the next spawning event within a month, score 2: large unpigmented (white or cream) eggs were clearly visible within the polyps, indicating that the colony would spawn within two months, score 3: small unpigmented (white or cream) eggs were visible within polyps, indicating the colony was unlikely to spawn for several months and score 4: no eggs were visible within polyps, indicating that the colony had recently spawned or would not spawn for many months.

### Laboratory samples

Following collection, colony samples were stored in a solution of 90% seawater and 10% formalin. The samples were decalcified in a solution of hydrochloric acid, formaldehyde (37%) and water. The initial solution was 5% HCl and 10% formaldehyde, but with a gradual increase in HCl from 5 to 10% over a period of weeks. After decalcification, the tissue samples were stored in 70% ethanol.

The tissue samples were then examined to determine the number and size of eggs in each polyp, as well as recording observations of mature testes, using a Leica MS205 stereoscope. Five polyps were dissected for each of the three branches sampled per colony. The polyps were sampled from the centre sections of the branch to avoid the sterile growing tips. All of the eggs within each polyp were counted and the maximal diameter measured using Leica Application Suite version 3.1 software. If no eggs were present a further 10 polyps were checked to confirm the results. We could not present the egg count data for *Favia stelligera* because total numbers of eggs per polyp were not counted for this species, but the spawning proportions and egg sizes have been included.

### Determining the month of spawning

The spawning month for each of the colonies sampled was determined using the following information: the in situ score given to the eggs, the presence of large testes, the proximity of the sample date to the predicted spawning date and the size of the eggs. Colonies with eggs that were small and unpigmented were predicted to spawn in the following spawning season, however these colonies could only be assigned a spawning season and not a particular month (6–8 months before spawning).

### Data analysis

To compare egg counts and egg sizes between mass and split spawning years, data from the mass spawning years (2008 and 2009) were pooled. Data were checked for normality using the Shapiro–Wilk test and for equality of variance using Levene’s test. Two sample *t*-tests were used to compare the means of egg counts per polyp and maximal egg size for each of the species between mass and split spawning years. Where data did not meet assumptions of normality, data were log transformed. If log transformed data still did not meet normality assumptions, the Wilcoxon two sample test was used to compare means. Of the 14 mean comparisons conducted, most (10) were normally distributed. The *A. spicifera* egg count data required log transformation before a *t*-test could be conducted. The *A. tenuis* egg count and *A. humilis* and *A. spicifera* egg size data did not meet assumptions of normality even after log transformation and were tested using the non-parametric Wilcoxon two sample test.

### Data availability

The data supporting the findings of this paper are available in the article and Supplementary Information. Raw data files are available from the authors upon reasonable request.

## Electronic supplementary material

Supplementary Information
