# Supplementary material for: Split spawning realigns coral reproduction with optimal environmental windows
Source: Nat Commun. 2018 Feb 19;9:718. doi: 10.1038/s41467-018-03175-2 (PMC5818648; doi:10.1038/s41467-018-03175-2)
Supplement: Supplementary file 1 — Supplementary Information [file 41467_2018_3175_MOESM1_ESM.pdf]

Supplementary Information

Supplementary Table 1. Summary of published reports of coral split spawning around the world.

Location	Year/s	Full Moon Date	Spawning Months	Reference
Great Barrier Reef	1981	First half of the month (13th Oct and 12th Nov)	Oct/ Nov	Harrison et al. 1984[3]
Great Barrier Reef	1984	First half of the month (10th Oct and 9th Nov)	Oct/ Nov	Willis et al. 1985[10]
Great Barrier Reef	1983	22nd Oct and 20th Nov	Oct/ Nov	Babcock et al. 1986[8]
Great Barrier Reef	1981-2005	First half of the month	Probably Oct/Nov*	Pharoah and Willis (unpublished) in Baird et al. 2009[26]
Coral Sea (GBR and Solomon Island)	1999	23rd November	Nov/ probably Dec*	Baird et al. 2000[27]
Scott Reef (WA)	1996	First week of the month (5th Mar and 4th Apr)	Mar/ Apr	Gilmour et al. 2009[23]
Oceanic Kimberley (WA)	Unspecified	Last week of the month	Mar/ Apr and Oct /Nov	Gilmour et al. 2016[17]
Dampier Archipelago (WA)	2007	First week of the month (4th Mar and 3rd Apr)	Mar/ Apr	Baird et al. 2011[24]
Houtman Abrolhos Islands (WA)	2013	Last week of the month (26th Feb and 27th Mar)	Mar/ Apr	Foster et al. 2015[28]
Amakusa (SW Japan)	2003	14th Jul and 12th Aug	Jul/Aug	Nozawa et al. 2006[29]
Thailand	2008	21st Feb, 21st Mar, 20th Apr	Feb/ Mar (northern sites, 14°N) and Mar/Apr (southern sites, 8°N)	Kongjandtre et al. 2010[30]
Singapore	2002	Last week of the month (27th Feb and 29th Mar)	Mar/Apr	Guest et al. 2005[16]
Moorea, French Polynesia	2002	21st Sep and 20th Oct	Early Oct/ late Oct, but only in two *Acropora* species	Carroll et al. 2006[31]
Caribbean	2002	22nd Aug and 21st Sep	Aug/Sep	Bastidas et al. 2005[32]
Persian Gulf	2008 and 2009	20th Apr and 20th May in 2008, 9th Apr and 9th May in 2009	Apr/May in both years	Bauman et al. 2011[33]

*One or both of the spawning months were not specified in the text.

Supplementary Table 2. Coral spawning observations and predictions at Scott Reef from 2007 to 2016. Some of the spawning dates were observed directly *in situ* or as spawn slicks on the water's surface, while others were inferred from egg presence/absence and egg pigmentation. If there was uncertainty with the number of days after the full moon (NAFM) that spawning occurred, then 8 nights was applied because corals at Scott Reef mass spawn 7-9 NAFM.

Year	Season	Spawning Month	Split or Mass	Full Moon Date	NAFM**	Spawn Date	Observed or Inferred
2007	Autumn	March	Split	4-Mar-2007	8	12-Mar-2007	Inferred
2007	Autumn	April	Split	3-Apr-2007	8	11-Apr-2007	Inferred
2007	Spring	October	Split	27-Sep-2007	9	5-Oct-2007	Inferred
2007	Spring	November	Split	26-Oct-2007	7	2-Nov-2007	Inferred
2008	Autumn	March	Mass	22-Mar-2008	8	30-Mar-2008	Inferred
2008	Spring	October	Mass	15-Oct-2008	7	22-Oct-2008	Observed
2009	Autumn	March	Mass	11-Mar-2009	8	19-Mar-2009	Inferred
2009	Spring	October	Mass	4-Oct-2009	8	12-Oct-2009	Observed
2010	Autumn	March	Split	1-Mar-2010	8	9-Mar-2010	Observed
2010	Autumn	April	Split	30-Mar-2010	7	6-Apr-2010	Inferred
2010	Spring	October	Split	23-Sep-2010	9	2-Oct-2010	Inferred
2010	Spring	October	Split	23-Oct-2010	7	30-Oct-2010	Inferred
2011	Autumn	March	Mass	20-Mar-2011	9	29-Mar-2011	Inferred
2011	Spring	October	Mass	12-Oct-2011	8	20-Oct-2011	Inferred
2012	Autumn	March	Mass	8-Mar-2012	9	17-Mar-2012	Observed
2012	Spring	October	Split	30-Sep-2012	9	9-Oct-2012	Observed
2012	Spring	November	Split	30-Oct-2012	9	8-Nov-2012	Inferred
2013	Autumn	March	Split	26-Feb-2013	9	7-Mar-2013	Observed
2013	Autumn	April	Split	27-Mar-2013	8	4-Apr-2013	Inferred
2013	Spring	October	Mass	19-Oct-2013	8	27-Oct-2013	Inferred
2014	Autumn	March	Mass	17-Mar-2014	8	25-Mar-2014	Inferred
2014	Spring	October	Mass	8-Oct-2014	8	16-Oct-2014	Observed
2015	Autumn	March	Split	6-Mar-2015	8	14-Mar-2015	Inferred
2015	Autumn	April	Split	4-Apr-2015	9	13-Apr-2015	Observed
2015	Spring	October	Split	28-Sep-2015	8	6-Oct-2015	Inferred
2015	Spring	November	Split	27-Oct-2015	8	4-Nov-2015	Inferred
2016*	Autumn	March*	Split*	23-Feb-2016	8	2-Mar-2016*	Inferred
2016*	Autumn	March*	Split*	23-Mar-2016	8	31-Mar-2016*	Inferred
2016*	Spring	October*	Mass*	16-Oct-2016	8	24-Oct-2016*	Inferred

* Mass coral bleaching and subsequent severe mortality in 2016 interrupted reproduction and no spawning or mature eggs were observed, however we have included the predicted spawning dates.
** NAFM = Nights After Full Moon.

Supplementary Table 3. Sample sizes (n) and the number of coral colonies spawning in March versus April in 2008, 2009 and 2010, for the proportions presented in Figure 4.

Species	2008 n	2008 March	2008 April	2009 n	2009 March	2009 April	2010 n	2010 March	2010 April
Acropora gemmifera	21	21	0	39	39	0	16	5	11
Acropora humilis	14	14	0	71	70	1	25	7	18
Acropora hyacinthus	11	11	0	32	31	1	5	3	2
Acropora microclados	5	5	0	31	31	0	3	1	2
Acropora polystoma	3	3	0	40	39	1	15	4	11
Acropora spicifera	187	187	0	128	128	0	16	3	13
Acropora tenuis	12	12	0	46	46	0	17	3	14
Favia stelligera	9	9	0	16	16	0	7	5	2

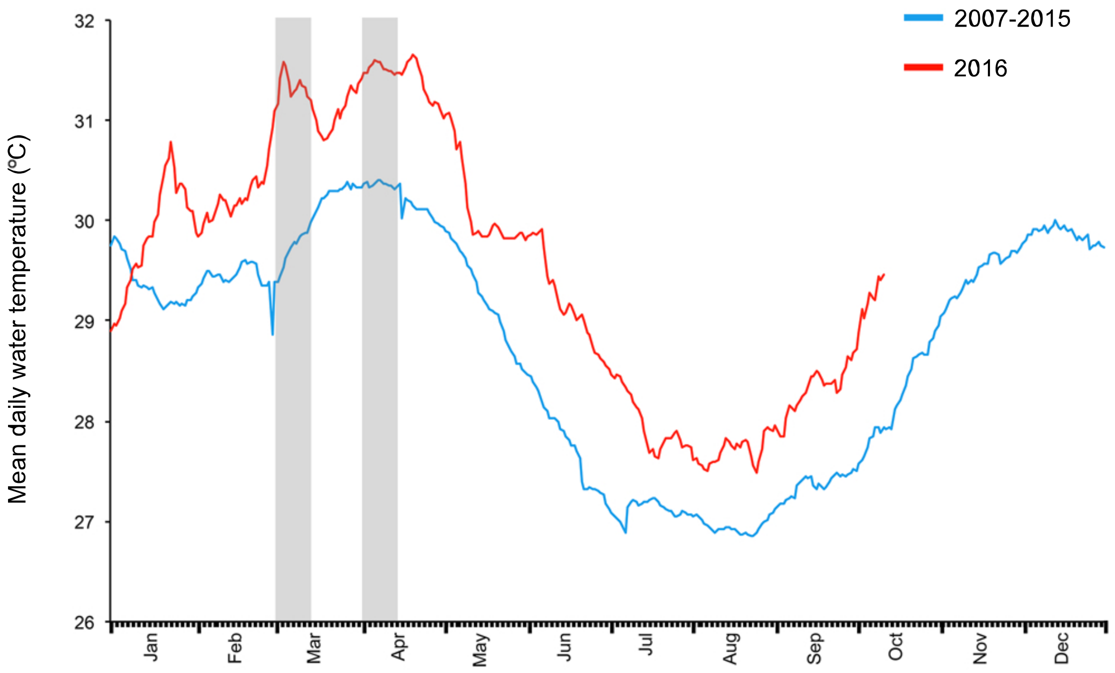

Supplementary Figure 1. Daily mean seawater temperatures at Scott Reef across all sites (temperature loggers at approximately 6 m water depth) for 2007-2015 and 2016. Predicted spawning dates for autumn 2016 highlighted in grey.

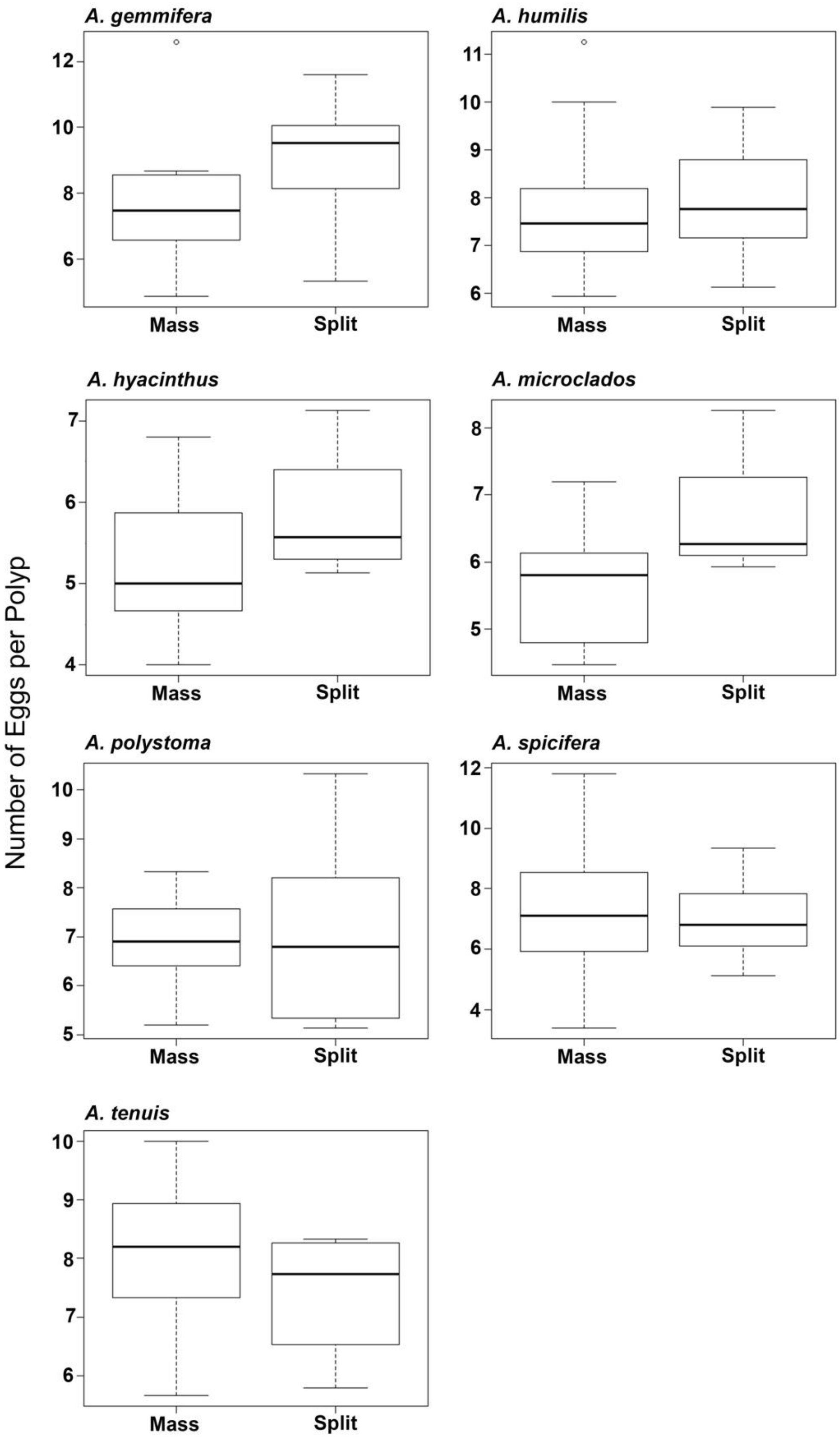

Supplementary Figure 2. Number of eggs per polyp in mass spawning (2008 and 2009) compared to split spawning (2010) years for 7 *Acropora* coral species at Scott Reef. There were no significant differences between split and mass spawning years. Mean comparisons were conducted using two sample t-tests, except in the case of *A. tenuis*, which was tested using the Wilcoxon two sample test.

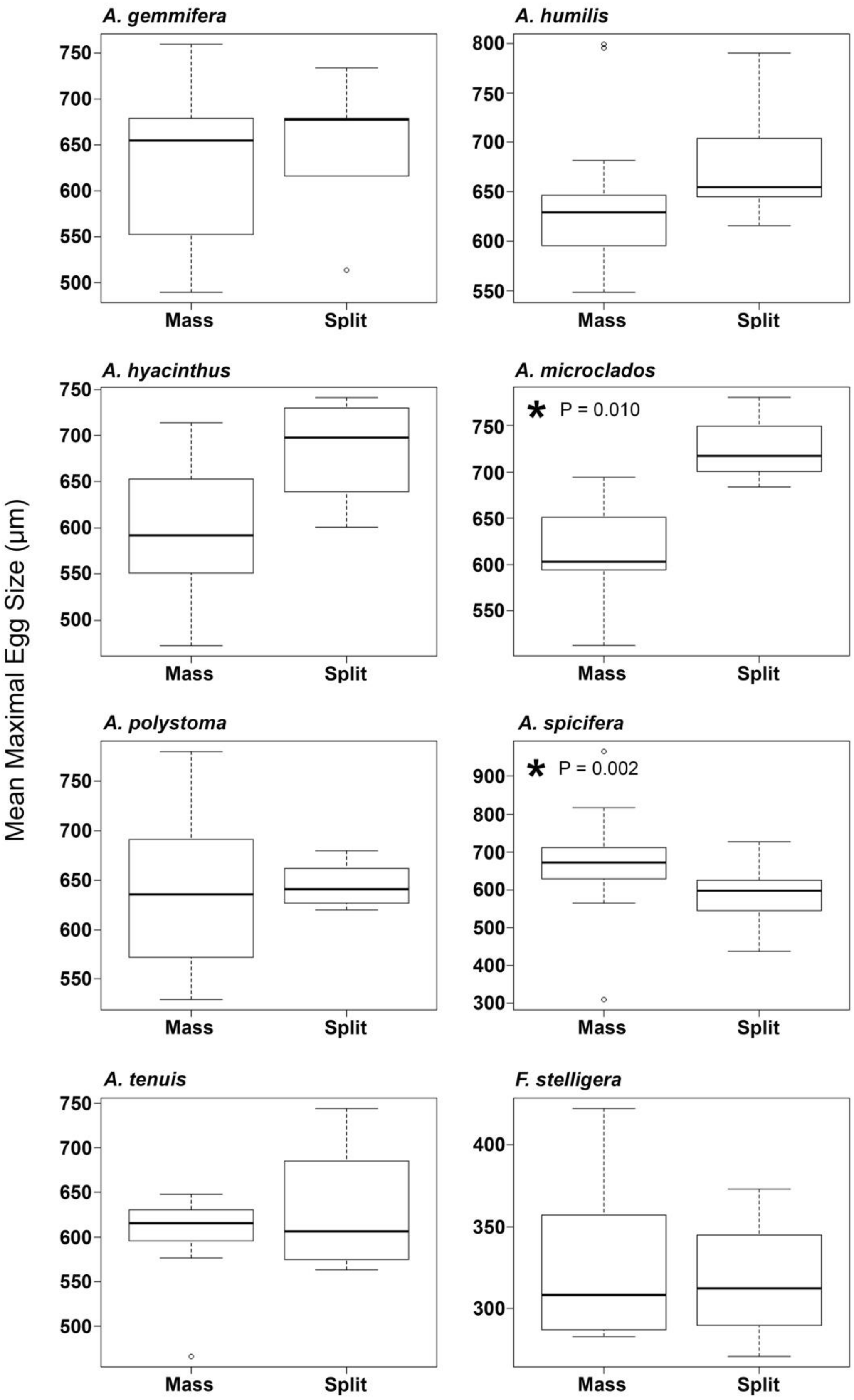

Supplementary Figure 3. Mean maximal egg sizes in mass spawning (2008 and 2009) compared to split spawning (2010) years for 8 coral species at Scott Reef. Mean comparisons were conducted using two sample t-tests, except in the case of *A. humilis* and *A. spicifera*, which were tested using the Wilcoxon two sample test. Significant differences ($P < 0.05$) are indicated with *

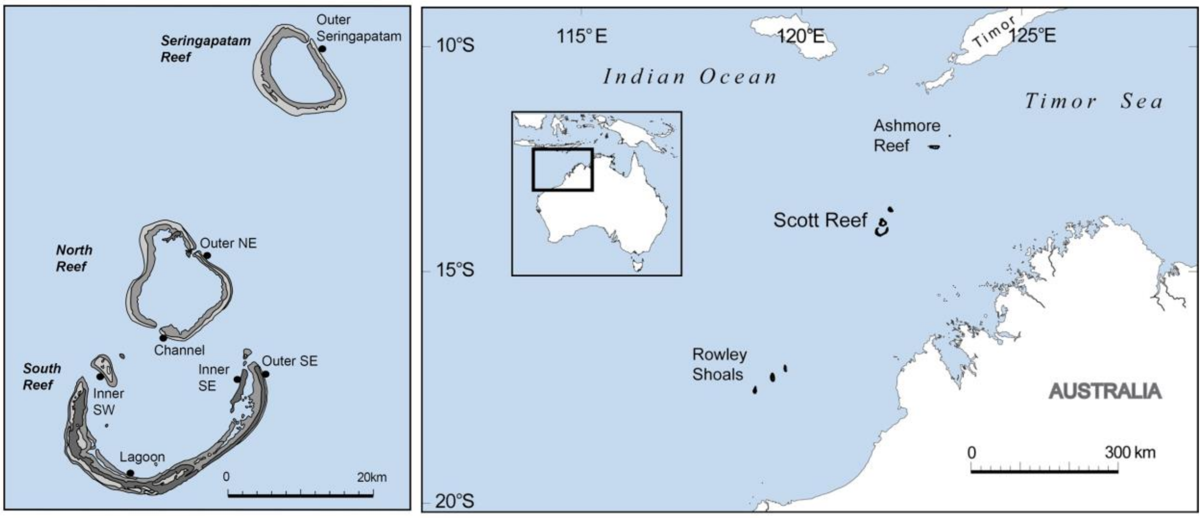

Supplementary Figure 4. Site map of Scott Reef off the northwest shelf of Western Australia. The map was generated by co-author James Gilmour using the software Adobe Illustrator.

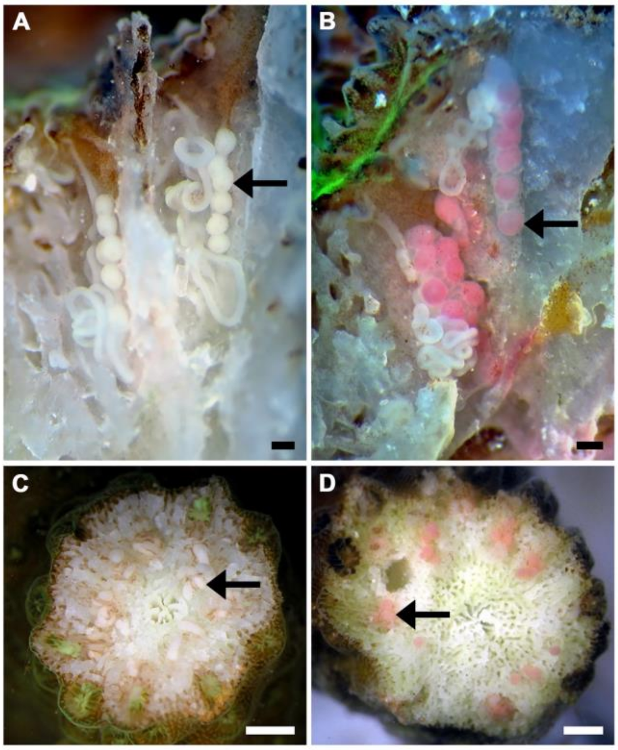

Supplementary Figure 5. Stage 1 and 2 of coral egg development for *in situ* scoring. Corals scored 2 have large unpigmented white or cream eggs (A and C) and corals scored 1 have large pigmented red or pink eggs (B and D). **A**) *Favites* scored 2, scale bar = 300 μm, **B**) *Favites* scored 1, scale bar = 400 μm, **C**) *Acropora* score 2, scale bar = 2 mm, **D**) *Acropora* score 1, scale bar = 2 mm. Photographs taken by A. Heyward and J. Gilmour.
